# The Time Course of Cardiorespiratory Adaptations to Rowing Indoor Training in Post-Menopausal Women

**DOI:** 10.3390/ijerph20043238

**Published:** 2023-02-13

**Authors:** Renata Cardoso Araujo, Gabriel Dias Rodrigues, Luana Farinazzo Ferreira, Pedro Paulo da Silva Soares

**Affiliations:** 1 Post-Graduation Program in Cardiovascular Sciences, Fluminense Federal University, Niterói, Rio de Janiero 24033-900, Brazil; 2 Department of Clinical Sciences and Community Health, University of Milan, 20122 Milan, Italy; 3 Department of Physiology and Pharmacology, Fluminense Federal University, Rio de Janiero 24210-130, Brazil

**Keywords:** rowing, whole-body exercise, maximal oxygen uptake, aging, heart rate

## Abstract

Background: Post-menopausal women have impaired cardiorespiratory responses to exercise compared to young women. Exercise training may counterbalance impairments, but the time-dependent effects of exercise training remain unclear. The current study aims to investigate the effects of rowing training on maximal aerobic capacity and time-course cardiorespiratory adaptations in older women. Methods: Female participants (*n* = 23) were randomly allocated to the experimental group (EXP; *n* = 23; 66 ± 5 years old) enrolled in rowing exercise training and control group (CON; *n* = 10; 64 ± 4 years old). The cardiopulmonary exercise test (CET) was performed in a cycle ergometer pre- and post-interventions. Oxygen uptake (VO_2_), stroke volume (SV), cardiac output (CO), and HR were recorded during CET and analyzed at the peak of the exercise. HR was monitored during exercise recovery, and the index of HRR was calculated by ΔHRR (HR_peak_—HR one-minute recovery). Every two weeks, Rowing Stepwise Exercise (RSE) in a rowing machine was performed to track specific adaptations to the exercise modality. HR was continuously recorded during RSE and corrected for the average power of each step (HR/watts). The rowing training protocol consisted of three weekly sessions of 30 min at an intensity corresponding to 60–80% of peak HR for ten weeks. Results: Rowing exercise training increased VO_2_, SV, and CO at the peak of the CET, and ΔHRR. Increased workload (W) and reduced HR response to a greater achieved workload (HR/W) during RSE were observed after six weeks of training. Conclusions: Rowing exercise training is a feasible method to improve cardiorespiratory performance, vagal reactivation and heart rate adjustments to exercise in older women.

## 1. Introduction

Aging leads to a progressive reduction in physical capacity, which is classically represented by the reduction in peak oxygen consumption (VO_2peak_) [1]. In addition, elevated heart rate (HR) responses during submaximal physical effort may also be observed in seniors, which could be attributed as a maker of poor cardiovascular function [2,3]. In contrast, physically active individuals show a lower decline over the years in VO_2peak_ than sedentary individuals [4] that could be influenced by exercise intensity, volume, and frequency [5]. Among the possible countermeasures to attenuate this age-related decline in the cardiorespiratory system, an integrative approach that combines resistive and endurance loading elements have been recommended in current guidelines [6]. In addition, the combination of diet and exercise programs may provide an additional benefit to the older population. In particular, the Mediterranean diet produces a further microvascular vasodilatory improvement in post-menopausal women, highlighting a putative intervention for further cardiovascular risk reduction [7].

Rowing is an Olympic sport modality characterized by high aerobic and resistance components that involve large muscle mass and may produce significant gains in physical capacity [8]. In a century-long follow-up study, the overall mortality of French Olympic rowers was reduced by 42% compared to the expected mortality for the general population [9]. It is remarkable that rowing exercise practice is able to promote health benefits for the general population [10]. However, it is unclear if the higher life expectancy in rowers is influenced by the selection of individuals more than the chronic positive effect of rowing training per se [8].

Despite most of the studies that investigated the rowing training effects being conducted with master athletes, it was demonstrated that rowing is a feasible training modality to prevent age-related muscle wasting and weakness [11], to improve muscle power, aerobic capacity [12] and endothelial function [13] in the older general population. Moreover, rowing training preserved skeletal muscle structure and function in key-antigravity muscles after five weeks of bed rest [14]. Regarding bone structure, master rowers had a greater bone mineral density than age-matched non-athletes, highlighting the benefit of systematic rowing training to prevent osteoporosis [15]. Due to its integrated cardiovascular benefits, indoor rowing exercises have been easily implemented in the training program of young and aged patients [16], and it may be a primary component of an integrated exercise prescription and countermeasure benefiting astronauts and patients affected by chronic deconditioning [14].

Remarkably, rowing is associated with few injuries compared to other sports because, in part, eccentric muscle contractions, the principal causes of the most common connective tissue injuries, are not frequent during rowing activity [8]. Further, rowing may benefit the brain, as described in a recent review [8]. Namely, rowing training reduced the cerebral metabolism demand (cerebral metabolic rate for oxygen) during exercise and increased the release of BDNF (brain-derived neurotrophic factor) [17]. Thus, rowing is within a rationale of exercise modality that may contribute to health promotion in the older population, even without extensive current literature.

In the older population, traditional aerobic physical training (e.g., jogging and cycling) reduces heart rate and blood pressure responses at rest and during submaximal exercise, even with low volume and intensity of exercise training [18,19,20], and increased vagal modulation to the heart at rest [21] as post-effort vagal reentry in older women [22]. Regarding the training volume, the current literature suggests an improvement in physical capacity and cardiovascular responses during aerobic training protocols of low to moderate intensity between 6 and 16 weeks in sedentary elderly [23]. Although aerobic exercises improve aerobic capacity in older adults, the time course of these adaptions from the whole body and multicomponent training such as rowing is still unclear. Since “one size does not fit all”, more options for exercise training (i.e., different motor tasks, target intensity, frequency and volume) should be investigated regarding their potential benefits for the general health and physiological outcomes in post-menopausal women.

The present study was undertaken to test the hypothesis that ten weeks of indoor rowing training increases maximal physical capacity and post-exercise heart rate recovery in post-menopausal women. Moreover, we expected an increased workload during rowing exercises with a lower chronotropic demand during the exercise training session during the protocol period. This study aims to investigate the effects of rowing exercise training on maximal aerobic capacity and time-course cardiorespiratory adaptations in post-menopausal women.

## 2. Materials and Methods

### 2.1. Sample

The sample was selected from a database of participants aged above 60 years old at the Fall Prevention Exercise Program at the Institute of Physical Education at Federal Fluminense University. The exclusion criteria were the use of any medication that could influence the outcome of the study (cardiovascular, neurological, respiratory, muscular/osteoarticular or metabolic diseases), symptoms or altered responses to a clinical exam and/or exercise test, participation in any other exercise training program, and were not naïve to rowing modality. The volunteers were restricted from caffeine consumption on the day of the test, had their last meal 2 h before the protocol and avoided physical exercise 24 h before the protocol. The temperature of the laboratory was controlled between 22 °C and 24 °C during experimental tests.

The protocol was approved by the Ethics Committee of the Fluminense Federal University (CEP/UFF) (2.172.240) following the Declaration of Helsinki and all volunteers signed a consent form before all procedures.

### 2.2. Experimental Protocol

**Figure 1:**
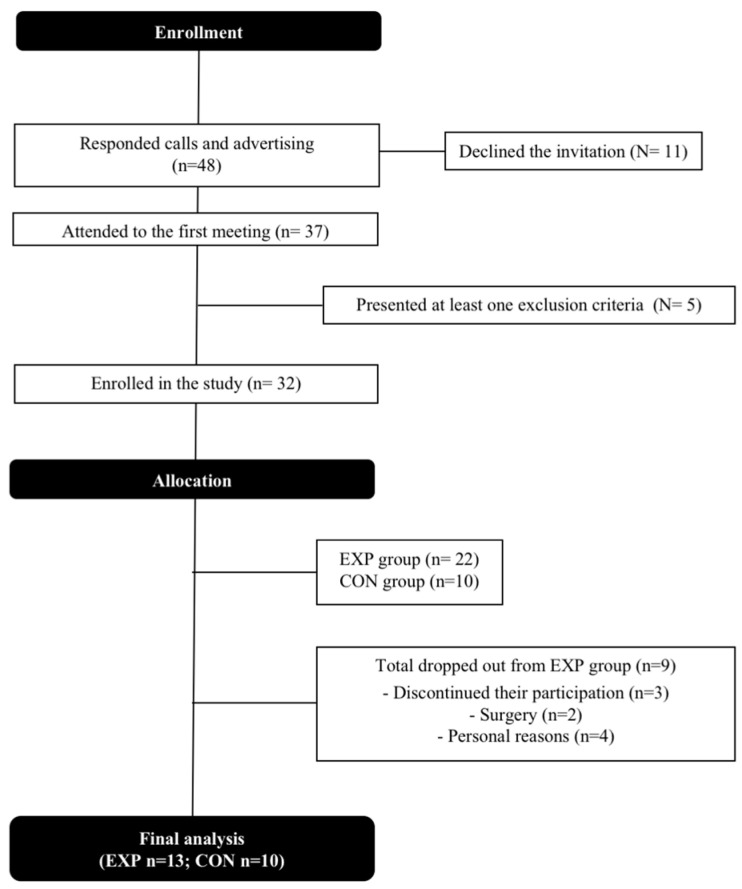
Flowchart of the study. EXP: experimental group; CON: control group.

Firstly, among the 48 older adults who were contacted, 11 declined the invitation. Thus, 37 participants underwent the cardiovascular pre-participation screening, which consisted of (1) a clinical exam, (2) an electrocardiogram (ECG) at rest, and (3) a cardiopulmonary exercise test (CET). After that, 32 participants (5 met at least one exclusion criterion) were randomized into the experimental group (EXP; *n* = 22), enrolled in the rowing exercise training protocol, and the control group (CON; *n* = 10), who did not engage in any exercise training program. The randomization was conducted by coin toss for every pair of age-matched participants. The author that analyzed the data was blinded to the group’s allocation. Before the end of the protocol, nine individuals dropped out of the EXP group for personal or medical reasons (Figure 1), and thirteen completed the training protocol.

### 2.3. Cardiopulmonary Exercise Test (CET)

CET started with three minutes of warming-up with gradual increments of 10 to 15 w·min^−1^ (Ergo Control Software; Inbramed^®^, Porto Alegre RS, Brazil), and cadence between 50 and 60 rpm in an electromagnetic resistance cycle ergometer (Inbramed^®^. Porto Alegre RS, Brazil) employing a ramp protocol until voluntary exhaustion. Workload increments were calculated according to the following equation: (increment in W/minute for women = [(height in cm − age in years) × 14] − [150 + (6 × body weight in kg)]/100) [24].

The oxygen consumption (VO_2_), minute ventilation (VE), and respiratory coefficient (RQ) were recorded using a gas analyzer (VO2000 Inbramed^®^, Porto Alegre RS, Brazil) every 20 s during CET. Cardiac output (CO), systolic volume (SV) and heart rate (HR) were recorded using a non-invasive transthoracic bioimpedance (PhysioFlow Lab1, Manatec Biomedical^®^, Poissy, France), 12-lead ECG (Wincardio, Micromed^®^, São Paulo SP, Brazil) was continuously recorded during CET. Blood pressure (BP) was obtained using the auscultatory method every minute (Premium, São Paulo SP, Brazil). The peak HR was considered the highest value of HR before the end of the CET. The index of post-exercise heart rate recovery (HRR) was calculated using ΔHR (HR_peak_—HR one-minute recovery) [25]. 

### 2.4. Rowing Stepwise Exercise

Rowing Stepwise Exercise (RSE) was a step-incremented adapted protocol [12] in a rowing machine (Concept II Model E, Morrisville VT, USA), and repeated every two weeks of training, until the eighth week, to track specific adaptations to exercise modality. RSE consisted of three steps of five minutes each, starting from a target workload of 20 watts, the second of 40 watts and the third step of 60 watts. If the target workload was not reached, the volunteer was encouraged to finish the step (5 min), and the obtained average workload was recorded. HR was continuously recorded during RSE and corrected for the average power of each step (HR/watts), to consider the time-course changes for individual physiological responses.

### 2.5. Rowing Exercise Training Protocol

The exercise training protocol consisted of three weekly sessions on a rowing machine (Concept II Model E, Morrisville, VT, USA) for ten weeks. Participants exercised for 30 min (including a 5-min warm-up and 5-min cooldown), and intensity corresponded to 60–80% of peak HR obtained by the CET at baseline (1). Heart rate (Bioharness, Zephyr, Minneapolis MN, USA), workload, distance, stroke rate, and frequency of training (at least 75% of 30 sessions) were monitored. The training prescription was divided into two parts: (1) familiarization sessions with specific exercises to learn the modality technique and (2) regular sessions. The regular session was divided into four parts: (1) stretching (the main muscles of the body, better definition), (2) warming up with educational exercises at 40–50% of HR_peak_, (3) main phase at 60–85% of HR_peak_ and (4) cooldown at 40–50% of HR_peak_. The main phase had four rounds of five minutes of rowing with a passive recovery of 1 min between the rounds.

### 2.6. Statistical Analysis

The Shapiro–Wilk test was applied to evaluate the normality of the data. The required sample size was considered an average statistical power equal to or higher than 0.80 for the main outcome variable VO_2peak_. The sample enrolled provided a statistical power of 0.99. Sample size (n) is computed as a function of the required power level (1-β), the considered significance level (α < 0.05), and the population effect size to be detected with probability 1-β [26]. Once the prior sample size was considered satisfactory, a post hoc analysis was conducted to confirm these assumptions.

In addition, the effect size (ES) was used to calculate the magnitude of the differences. The ES was computed as Cohen’s d (t-test) and f (ANOVA) statistics and interpreted as following: trivial (0–0.2), small (0.2–0.6), moderate (0.6–1.2), large (1.2–2.0), and very large (2.0–4.0) [27].

For the comparison between cardiovascular responses and metabolic variables before and after training, Two-way ANOVA for repeated measurements in the time factor with Sidak’s post hoc was used. The level of significance of α ≤ 0.05 was adopted. The descriptive statistics were mean and standard deviation. The software used was GraphPad Prism version 8.0 (GraphPad Software Inc, Boston, MA, USA).

## 3. Results

### 3.1. Sample

Thirty-seven volunteers were selected. From the first sample, five of them showed alteration in the screening tests, and nine volunteers started the training protocol but did not complete it for medical or personal reasons. Twenty-three completed the protocol and all procedures, and were allocated into two groups, EXP and CON, after random selection. The details of the flowchart are shown in Figure 1.

The participants were not different by age (EXP 66 ± 5 vs. CON 64 ± 4 years old; *p* = 0.50) and body mass index (EXP 26.95 ± 3.20 vs. CON 26.98 ± 3.35 kg/m^2^; *p* = 0.29) at baseline. There were no significant differences in participants’ characteristics and hemodynamic variables at rest between the groups at baseline and post-intervention (Table 1).

### 3.2. Maximal Cardiorespiratory Fitness following Rowing Training

Maximal aerobic capacity (VO_2peak_), SV_peak_ and CO_peak_ increased post-rowing training in the experimental compared to the control group. VE_peak_, HR_peak_ and RQ_peak_ did not change post-rowing or control interventions. However, HRR increased for the experimental group post-intervention compared to the baseline. These results are summarized in Table 2.

### 3.3. Heart Rate and Workload Responses to Rowing Stepwise Exercise after Training

**Figure 2:**
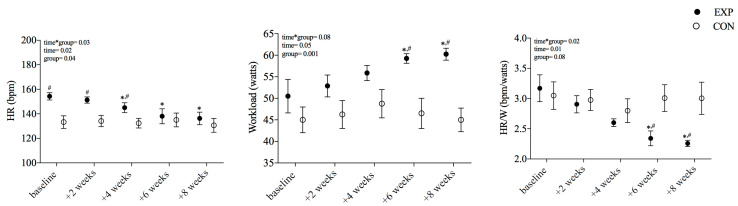
Workload and heart rate responses to rowing stepwise exercise during rowing exercise training. A. Workload (watts) and B. heart rate adjusted for power (bpm/watts) for every week of training. Measures were obtained during the last stage of the Rowing Stepwise Exercise test. ANOVA with repeated measures in the time factor. Sidak’s post hoc. Mean ± SD. EXP: experimental group; CON: control group. * Differences from baseline. # Differences between groups.

The workload acquired at the last step of the rowing exercise was increased following six and eight weeks of the training intervention in experimental compared to baseline. The HR response to RSE was higher at baseline and after two weeks in the EXP group compared to the CON group. After four weeks, the HR response to exercise was reduced only in the EXP group and remained lower until the end of the protocol. The ratio between HR and W (HR/watts) showed reduced HR responses to increased workloads achieved during the third step of RSE after six and eight weeks of training. These results are shown in Figure 2. Lastly, it is noteworthy that HR achieved at the last step of RSE at the baseline was not statistically different from the HR_peak_ during CET at bicycle (CET 162 ± 15 vs. RSE 154 ± 8 bpm; *p* = 0.22).

## 4. Discussion

The major findings from the current study are the following: (1) Rowing exercise training increased oxygen uptake, stroke volume and cardiac output at the peak of the cardiopulmonary exercise test in post-menopausal women; (2) Rowing training improved post-exercise heart rate recovery suggesting increased vagal reentry; (3) A diminished heart rate response and increased workload during rowing stepwise exercise were observed following rowing exercise training. Thus, post-menopausal women who underwent rowing training improved the specific work capacity of the exercise modality with better adjustments in heart rate response.

VO_2peak_ reflects the workings of the cardiovascular, respiratory and neuromuscular systems during maximal exercise. VO_2peak_ is the gold-standard measure of health and physical fitness [28], and it was associated with both morbidity and mortality risks [29]. VO_2peak_ is decreased with age, but could be increased in elders with the same magnitude as observed in young individuals following endurance exercise training [4]. VO_2peak_ improvements from exercise training are determined by central (stroke volume and cardiac output), and peripheral (arteriovenous difference) adaptations. The CO_peak_ is the major component of the age-related decline in VO_2peak_. Impaired CO responses to exercise in older adults could be a consequence of some factors: (1) decreased systolic and diastolic function; (2) increased vascular stiffness and aortic impedance; (3) decreased inotropic and chronotropic sensitivity [30].

Despite the physiological declines attributed to aging, a physically inactive lifestyle also contributes to reduced cardiac function (and thus in VO_2peak_) with advancing age [1]. Age-related decrements in CO_peak_ could be reversed by endurance training in older adults, especially with substantial increment in SV during exercise [31]. Otherwise, underlying mechanisms involved in the training-induced improvements in VO_2peak_ could be different between sexes, as older women are more dependent on peripheral adaptations (i.e., increased arteriovenous difference) [32].

The practice of rowing can promote health benefits for the general population [10]. Despite that, few studies investigated physiological adaptions by rowing training in older adults [11,12,13]. Rowing prevented age-related muscle wasting and weakness [12] and improved endothelial function [13]. A case-controlled study showed a higher total and regional body mineral density in master rowers compared to age-matched non-athletes [15]. In accordance, a recent meta-analysis showed that both aerobic and resistance training increased body mineral density in post-menopausal women [33].

Regarding cardiorespiratory fitness, rowing-trained older men showed greater VO_2peak_ and lower limb muscular strength than the sedentary age-matched group. In this report, the trained group was composed of men who practiced rowing at least twice per week for five years or more. Thus, the lower reduction in VO_2peak_ observed in older rowers seems to be influenced by a physically active lifestyle with advancing age, but not an effect from a rowing training intervention in older sedentary adults [10].

From our results, rowing training is a feasible modality to enhance VO_2peak_ in older women. In addition, the improved VO_2peak_ is accompanied by higher SV_peak_ and CO_peak_. To our knowledge, this is the first study to demonstrate a positive effect of rowing training in healthy post-menopausal women who were naïve to this sports modality and were not enrolled in any exercise program at the moment of the protocol.

Results from previous studies suggested that low-volume high-intensity interval training provoked a rapid improvement in VO_2peak_ compared to higher-volume moderate-intensity continuous training in post-menopausal women [34]. In addition, a recent study [35] compared two programs of interval cycling training with moderate and heavy intensities for twelve weeks in post-menopausal women. The authors reported that only the heavy exercise increased VO_2peak_ and gas exchange threshold. Following these previous findings, our results suggest that rowing training induces rapid improvements in workload and submaximal HR to exercise (Figure 2). Indeed, HR response to exercise overcame the target HR, but six weeks of training reduced the HR response to a higher workload. The increased workload during the third step of RSE should be first attributed to mechanical factors and technique, but the HR adjusted to workload suggests a classical adaptation to aerobic training. Thus, the time-dependent effects of exercise help to understand how much training volume is needed to adapt the cardiorespiratory function in this particular population.

In the current study, maximal heart rate did not change in both experimental and control groups, as observed by others [22,36]. However, rowing training improved heart rate recovery after cardiopulmonary exercise (Table 2). Heart rate recovery is a powerful predictor of overall mortality, independent of workload, and the presence of cardiovascular diseases in the young and the older population [25].

In older hypertensive women, a walking aerobic training program (12 weeks) accentuated the decrement in heart rate after a treadmill cardiopulmonary exercise test [22]. In addition, older patients who completed phase II of a cardiac rehabilitation program, improved post-exercise heart rate recovery [37]. Vagal reactivation seems to be the main physiological mechanism responsible for faster heart rate recovery in older adults [38]. Moreover, heart rate recovery 1 min after exercise is dependent on the mode (i.e., lower in the cycle than treadmill) and intensity of exercise in healthy and heart failure subjects [39].

Heart rate recovery is also associated with the measurements of 24 h heart rate variability in older men [40], and with vagal-mediated indexes of heart rate variability increased by exercise training in healthy young subjects [41]. Older and young subjects matched by fitness levels showed similar heart rate recovery, despite distinct results between untrained young and older subjects (i.e., greater HRR in the younger group) [42]. Recently, no differences were found in heart rate recovery between older adults with high and low physical fitness [43]. Thus, the impact of physical fitness and aging on the autonomic control of the heart is still a matter of controversy.

The aging process reduces heart rate responses during light [2], moderate [3], and high [43] levels of exercise. Indeed, older adults had a reduced cardiac vagal withdrawal and sympathetic drive to light cycle exercise than young subjects [2]. As regards the training effects, maximal workload, VO_2_ and submaximal heart rate response to exercise were improved following increased daily physical activity in the elderly [18,21]. Indeed, improvements in the submaximal responses to exercise training have noteworthy importance to the older population because of the accomplishment of daily activities [20].

Lastly, our data showed an increment in absolute workload reached at the last stage of the rowing stepwise exercise post-training in the experimental compared to the control group, with a lower heart rate response to the increased workload. These findings suggest improvements in the specific work capacity for the exercise mode (rowing) with a lower cardiac demand (heart rate) by rowing training in post-menopausal women.

## 5. Limitations

The current study has two main limitations: (1) differences in the sample size between groups; (2) the target HR for each rowing exercise section was not calculated by the HR_peak_ achieved during the CET at the bicycle ergometer. Although it was not ergometer-specific, the achieved HR at the last stage of RSE and the HR_peak_ during CET were similar (please see results section, subtitle 3.3). In addition, we opted for the bicycle ergometer for two main reasons: (1) it should be safer to perform the pre-participation screening for this population; and (2) to our knowledge, there is not a validated protocol for maximal tests using the rowing ergometer in non-athletes, namely older post-menopausal women. Finally, the sample size seems to be small, but the effect size of the rowing training was robust enough to provide reliable results.

## 6. Conclusions

Rowing exercise training is a feasible and efficient method to increase peak oxygen uptake, stroke volume and cardiac output, and to improve vagal reactivation in older women. In addition, post-menopausal women who underwent rowing training improved their specific work capacity in the exercise mode with better adjustments in heart rate response. In conclusion, older women can row and present cardioprotective adaptations from training, and rowing exercises should be encouraged and introduced in physical activity programs for healthy aging.

## Figures and Tables

**Table 1:** Participants’ characteristics and hemodynamic variables at rest in experimental and control groups at baseline and post-intervention.

	CON (*n* = 10)		EXP (*n* = 13)		*p*-Value		
	Baseline	Post-Intervention	Baseline	Post-Intervention	Int.	Time	Group
Total body mass (kg)	64.86 ± 8.95	64.88 ± 9.06	60.37 ± 11.75	60.40 ± 11.95	0.98	0.92	0.33
SBP (mmHg)	121 ± 14	121 ± 15	124 ± 10	116 ± 10	0.03	0.04	0.61
DBP (mmHg)	77 ± 11	77 ± 12	76 ± 8	74 ± 8	0.48	0.48	0.33
HR (bpm)	82 ± 10	78 ± 10	76 ± 11	79 ± 10	0.20	0.77	0.38

BMI: body mass index; SBP: systolic blood pressure; DBP: diastolic blood pressure; HR: heart rate. EXP: experimental group; CON: control group. Two-way ANOVA to compare groups and time factors. Sidak’s post hoc. Mean ± SD.

**Table 2:** Cardiorespiratory responses during the maximum exercise test in experimental and control groups at baseline and post-intervention.

	CON (*n* = 10)		EXP (*n* = 13)		*p*-Value		
	Baseline	Post-Intervention	Baseline	Post-Intervention	Int.	Time	Group
VO_2peak_ (mL·kg^−1^·min^−1^)	18.2 ± 3.69	18.28 ± 3.41	18.06 ± 2.66	21.76 ± 3.22 *^,#,d^	0.01	0.01	0.22
VE_peak_ (L/min)	36.90 ± 4.65	37.66 ± 5.03	33.76 ± 6.76	35.40 ± 8.45 ^a^	0.07	0.34	0.47
RQ_peak_	1.06 ± 0.08	1.09 ± 0.12	1.08 ± 0.07	1.07 ± 0.07 ^a^	0.59	0.28	0.74
SV_peak_ (mL)	93.34 ± 16.96	87.72 ± 18.85	92.1 ± 18.08	103.84 ± 22.09 *^,#,c^	0.01	0.01	0.29
CO_peak_ (L/min)	12.50 ± 2.21	12.21 ± 2.07	14.87 ± 3.07	17.26 ± 4.14 *^,#,d^	0.01	0.01	0.01
HR_peak_ (bpm)	150 ± 10	153 ± 10	163 ± 15	164 ± 14 ^b^	0.67	0.03	0.13
HRR (bpm)	20 ± 2	20 ± 4	21 ± 1	27 ± 1 *^,c^	0.01	0.01	0.16

W = watts. VO2: oxygen uptake. HR: heart rate. CO: cardiac output. SV: stroke volume. HRR: HR peak—HR at one-minute post-exercise. Two-way ANOVA with repeated measures in the time factor and Sidak’s post hoc. The effect sizes are represented by letters “a”: trivial (0–0.2); “b”: small (0.2–0.6), “c”: moderate (0.6–1.2); and “d”: large (1.2–2.0). Mean ± SD. EXP: experimental group; CON: control group. * Differences from baseline. # Differences between groups.

## Data Availability

Data will be made available upon reasonable request.
